# High and Persistent HIV Seroincidence in Men Who Have Sex with Men across 47 U.S. Cities

**DOI:** 10.1371/journal.pone.0034972

**Published:** 2012-04-18

**Authors:** Marta-Louise Ackers, Alan E. Greenberg, Carol Y. Lin, Bradford N. Bartholow, Adrian Hirsch Goodman, Michael Longhi, Marc Gurwith

**Affiliations:** 1 Epidemiology Branch, Division of HIV/AIDS Prevention, National Center for HIV, STD, and TB Prevention, Centers for Disease Control and Prevention, Atlanta, Georgia, United States of America; 2 Quantitative Science and Data Management Branch, Division of HIV/AIDS Prevention, National Center for HIV, STD, and TB Prevention, Centers for Disease Control and Prevention, Atlanta, Georgia, United States of America; 3 VaxGen, Inc, Brisbane, California, United States of America; National Microbiology Laboratory, Canada

## Abstract

**Objective:**

To provide HIV seroincidence data among men who have sex with men (MSM) in the United States and to identify predictive factors for seroconversion.

**Methods:**

From 1998–2002, 4684 high-risk MSM, age 18–60 years, participated in a randomized, placebo-controlled HIV vaccine efficacy trial at 56 U.S. clinical trial sites. Demographics, behavioral data, and HIV status were assessed at baseline and 6 month intervals. Since no overall vaccine efficacy was detected, data were combined from both trial arms to calculate HIV incidence based on person-years (py) of follow-up. Predictors of seroconversion, adjusted hazards ratio (aHR), were evaluated using a Cox proportional hazard model with time-varying covariates.

**Results:**

Overall, HIV incidence was 2.7/100 py and was relatively uniform across study sites and study years. HIV incidence was highest among young men and men reporting unprotected sex, recreational drug use, and a history of a sexually transmitted infection. Independent predictors of HIV seroconversion included: age 18–30 years (aHR = 2.4; 95% CI 1.4,4.0), having >10 partners (aHR = 2.4; 95% CI 1.7,3.3), having a known HIV-positive male sex partner (aHR = 1.6; 95% CI 1.2, 2.0), unprotected anal intercourse with HIV positive/unknown male partners (aHR = 1.7; 95% CI 1.3, 2.3), and amphetamine (aHR = 1.6; 95% CI 1.1, 2.1) and popper (aHR = 1.7; 95% CI 1.3, 2.2) use.

**Conclusions:**

HIV seroincidence was high among MSM despite repeated HIV counseling and reported declines in sexual risk behaviors. Continuing development of new HIV prevention strategies and intensification of existing efforts will be necessary to reduce the rate of new HIV infections, especially among young men.

## Introduction

Currently human immunodeficiency virus (HIV) infects ∼48,000 persons in the United States annually, and 57% of HIV/AIDS cases are reported among men who have sex with men (MSM) [1], [2]. Although rates of HIV infection among MSM in the United States declined in the late 1980s and early 1990s, subsequent data indicated increases in HIV infections, sexually transmitted infections (STIs), and unsafe sexual practices among this population [3]–[15]. In general, estimates of HIV seroincidence among U.S. MSM are largely based on (1) small regional or local cohort studies which follow individuals for short time periods, (2) national HIV surveillance data from selected locations, and (3) cross-sectional studies or samples which use the BED HIV-1 assay within the serologic testing algorithm for recent HIV seroconversion (STARHS) to estimate HIV seroincidence. Large national MSM cohort studies remain the gold standard for HIV incidence estimates and can be utilized to provide data for a variety of age groups and geographic regions as well as valuable information on trends and risk factors for infection.

From 1998–2002, a large multi-site phase III HIV vaccine efficacy trial was conducted among 5108 high-risk HIV-1 negative MSM and 309 heterosexual women in North America and the Netherlands; U.S. MSM accounted for over 90% of study participants. Although the vaccine did not demonstrate efficacy [16], [17], enrollment and retention of high-risk participants were excellent [17], [18]. Thus, epidemiologic and behavioral data from this 36-month trial are useful for characterizing national trends in HIV incidence and identifying risk factors for HIV infection among U.S. MSM.

## Methods

### Study population

From June 1998 through October 1999, VaxGen, Inc. (Brisbane, CA) enrolled 4697 HIV-seronegative MSM into a 36-month, randomized (2∶1 vaccine∶placebo), double-blind, placebo-controlled phase III HIV-1 vaccine efficacy trial at 56 clinical trial sites in 47 cities in the United States. Enrolled MSM were healthy, 18–60 years of age, and reported having anal sex with ≥1 HIV positive or unknown serostatus male partner during the past year. Men in a monogamous relationship for ≥1 year with a HIV seronegative male partner or who reported injection drug use (IDU) in the preceding three years were excluded from enrollment. Recruitment strategies included advertising, outreach, and referrals from other cohort studies, from HIV-infected partners, and current trial participants.

HIV counseling and testing was conducted at enrollment, semiannually, and at participant request. Participants diagnosed with HIV infection during the trial were referred for medical care and social services. Nucleic acid testing (NAT) detected 13 HIV infections among MSM at enrollment; these men were discontinued from the trial resulting in 4684 MSM eligible for subsequent incidence analyses. Details of the trial baseline methods, study procedures, and efficacy results have been published elsewhere [17], [18].

### Ethics Statement

The vaccine study protocol was approved by the institutional review boards (IRB) of Saint Louis University, John Hopkins University School of Medicine, Fenway Community Health Center, Philadelphia Fight, AIDS Research Alliance, Louisiana State University Medical Center, University of California, Irvine, University of California, San Francisco, University of Washington, Hennepin County Medical Center, Mount Sinai Medical Center, the University of New Mexico, the University Medical Center of Southern Nevada, Abbot Northwestern Hospital, New York Blood Center, New York Medical Center, Howard Brown Health Center, Ohio State University, the University of Texas, Galveston, the University of California, Davis, Community AIDS Resource, Inc., University of Hawaii, the AIDS Research Consortium of Atlanta, Erie County Medical Center, Santa Clara County Public Health Department, Albany Medical College, New Jersey Community Research Initiative, Duval County Health Department, University Hospitals of Cleveland, University of Alabama at Birmingham, Community Hospitals Indianapolis, Wisconsin AIDS Research Consortium, University of Pittsburgh, Miriam Hospital, Nalle Clinic, and Memorial Hospital of Rhode Island, the Colorado Multiple IRB, the Western IRB, and the IRB of the Centers for Disease Control and Prevention (CDC). All participants provided written informed consent for trial participation.

### Data collection

At enrollment, a standardized interviewer-administered questionnaire was used to collect information on demographics, medical history, and reasons for trial participation. Data on STI diagnoses, concomitant medications, and sexual risk behaviors (i.e., unprotected receptive or insertive anal intercourse [UAI]) with partners by HIV-serostatus (negative, positive, and unknown) in the previous 6 months were collected at enrollment and 6 month intervals. Participants were also asked if they had injected drugs during the past six months, or if they had used marijuana/hash, poppers, crack or cocaine, amphetamines (e.g., crystal or speed), tranquilizers/sedatives (e.g., valium, Xanax, Klonopin, barbiturates, Quaaludes), hallucinogens (e.g., PCP, Special K, angel dust, acid, LSD, mushrooms, ecstasy), heroin, or drugs to enhance sexual performance (e.g., Viagra, Yohimbine). Two questionnaire items designed to assess beliefs about HIV vaccine trial participation that may increase HIV risk behavior, such as perceiving a high degree of protection against HIV if the vaccine was shown to be efficacious and perceiving vaccine treatment assignment were asked at enrollment (the first item) and at 12, 24, and 36 months (the second item).

### Laboratory Testing

Specimens were tested using an enzyme immunoassay (EIA) at a central laboratory; EIA reactive specimens were confirmed by Western Blot. If HIV seropositivity was detected, a specimen available at the time of the last negative EIA was tested by NAT (Procleix HIV-1 Discriminatory Assay, Gen-Probe, Inc., San Diego, CA) to determine viremia at an earlier visit.

### Statistical analysis

Incidence density was calculated by baseline characteristics based on person-years (py) of follow-up for HIV seronegative MSM who received at least one additional HIV test during the trial. The Breslow-Day test was used to assess heterogeneity in HIV incidence over time. Date of HIV infection was defined as the midpoint between the last negative and the first positive EIA or date of first positive NAT. A Cox proportional hazards model identified baseline variables predictive of loss-to-follow up. Baseline variables were evaluated to examine differences in reported risk behaviors between young (18–30 years of age) and older MSM using the Mantel-Haenszel [chi]^2^ test. Potentially predictive (p<.20) characteristics for HIV seroconversion identified in univariate Cox proportional hazards models with time-varying covariates updated every 6 months and forced inclusion of treatment arm assignment were assessed in a multivariate Cox proportional hazards model including partner serostatus, numbers of sexual partners, UAI, recreational drug use, age, and STI. Analyses were conducted using SAS (version 8.0; SAS Institute, Cary, NC).

## Results

### Participants

From June 1998 through October 1999, 4684 HIV-1 seronegative male trial participants enrolled at 56 clinical trial sites in 47 U.S. cities, encompassing 27 states, Washington, DC, and Puerto Rico. The median number of MSM enrolled per site was 75 (range, 5–303). Retention at 6, 12, 18, 24, 30, and 36 months was 96%, 92%, 89%, 87%, 85% and 82%, respectively. Loss-to-follow-up was independently associated with the following baseline characteristics: age 18–30 years (p<.0001), African American (p<.01) and Hispanic (p<.01) race, tranquilizer (p<.0001) and crack (p<.01) use. Retention was associated with popper (p<.01) and sexual performance enhancing drug (p<.05) use and completing college (p<.0001).

The baseline demographic and sexual risk behavioral characteristics of the U.S. MSM are presented in Table 1. The median age was 35 years, range 18–62; 25% were 18–30 years of age. Men were predominantly of white race/ethnicity, and most reported attaining at least one college degree. The majority of MSM were enrolled at clinical sites in the southern or western United States. The median number of male sex partners in the 6 months before enrollment was 5 (range 0–960 partners); 32% reported having ≥10 male partners. In the previous six months, 10% reported having a STI, and over half reported UAI. IDU was minimal (0.23%), however 61% reported non-injection recreational drug use (e.g., amphetamines, tranquilizers, poppers, cocaine, hallucinogens, and sexual performance enhancing drugs), and 87% reported alcohol use.

**Table 1:** US MSM HIV seroincidence (per 100 person-years) by participant demographic and behavioral characteristics at enrollment (n = 4684).

Characteristic	No. (%) N = 4684	No. SC N = 338	HIV Incidence/100 py (95% CI)
**Age category (years)**			
18–30	1153 (25)	117	3.7 (3.0, 4.5)
31–40	1917 (41)	144	3.0 (2.6, 3.5)
41–50	1192 (25)	61	2.0 (1.6, 2.6)
>51	422 (19)	16	1.6 (0.9, 2.5)
**Race/Ethnicity**			
White	4012 (86)	287	2.7 (2.4, 3.1)
Hispanic	319 (7)	22	2.8 (1.8, 4.3)
African American	173 (4)	10	2.3 (1.1, 4.4)
Asian	72 (2)	6	3.1 (1.2. 6.8)
Other	108 (2)	13	4.7 (2.5, 8.2)
**Education level**			
<High school	55 (1)	2	1.7 (.28, 5.5)
High school	1678 (36)	132	3.2 (2.7, 3.9)
College	2950 (63)	204	2.6 (2.3, 3.0)
**Geographic region of enrollment**			
Midwest	912 (19)	56	2.4 (1.8, 3.1)
Northeast	873 (19)	60	2.6 (2.0, 3.4)
South	1490 (32)	105	2.9 (2.4, 3.5)
West	1409 (30)	116	3.2 (2.6, 3.8)
**Sexual risk behavior in previous 6 months**			
Having >1 female sex partner	221 (5)	10	1.9 (0.9, 3.5)
Having >1 HIV-infected male partner	2064 (44)	193	3.5 (3.0, 4.1)
Any UAI	2713 (58)	254	3.6 (3.2, 4.1)
Any receptive UAI	1727 (37)	195	4.4 (3.8, 5.0)
Any insertive UAI	2212 (47)	202	3.9 (3.3, 4.5)
UAI with positive/unknown serostatus partner	1838 (39)	189	3.9 (3.4, 4.6)
Receptive UAI with positive/unknown serostatus partner	944(20)	123	4.9 (4.0, 5.8)
Insertive UAI with positive/unknown serostatus partner	1447 (31)	156	4.1 (3.5, 4.8)
History of STI	450 (10)	31	3.8 (2.6, 4.4)
**History of recreational drug/substance use in previous 6 months**			
Injection drug use	11 (.23)	1	2.8 (2.5, 3.1)
Amphetamines	417 (9)	62	5.0 (3.8, 6.4)
Crack	92 (2)	13	5.2 (2.7, 8.8)
Cocaine	575 (12)	58	4.0 (3.1, 5.2)
Poppers	1458 (31)	168	4.4 (3.8, 5.2)
Tranquilizers	629 (13)	56	3.6 (2.7, 4.6)
Sexual performance enhancing drugs	590 (13)	59	3.7 (2.8, 4.8)
Hallucinogens	603 (13)	70	4.6 (3.6, 5.9)
Alcohol	4067 (87)	617	2.8 (2.5, 3.1)

NOTE: Not all columns add up to 4684 due to non-response. SC, seroconverters; UAI, unprotected anal intercourse.

Young men (≤30 years of age) were less likely than older men to report a male HIV-infected partner at enrollment, but were more likely to report any receptive UAI 41% vs. 35% (p = <.001). They were also less likely than older men to report use of poppers or sexual performance enhancing drugs, but more likely to report using marijuana, amphetamines, cocaine, and hallucinogens. Furthermore, over the course of the study, amphetamine use significantly increased among young men (from 11% at baseline to 16% at 36 months; p = <.01). The use of other recreational drugs did not differ by age or time.

### HIV Incidence

A total of 338 HIV infections were detected among the 4684 MSM during the trial. Fifty-one sites reported ≥1 case of HIV infection. Participants who became infected with HIV were predominantly white (85%); 60% reported a college degree. The median age at the time of infection was 36 years (range, 20–62 years), and the median time to infection after study enrollment was 16 months (range, .6–35 months).

**Figure 1:**
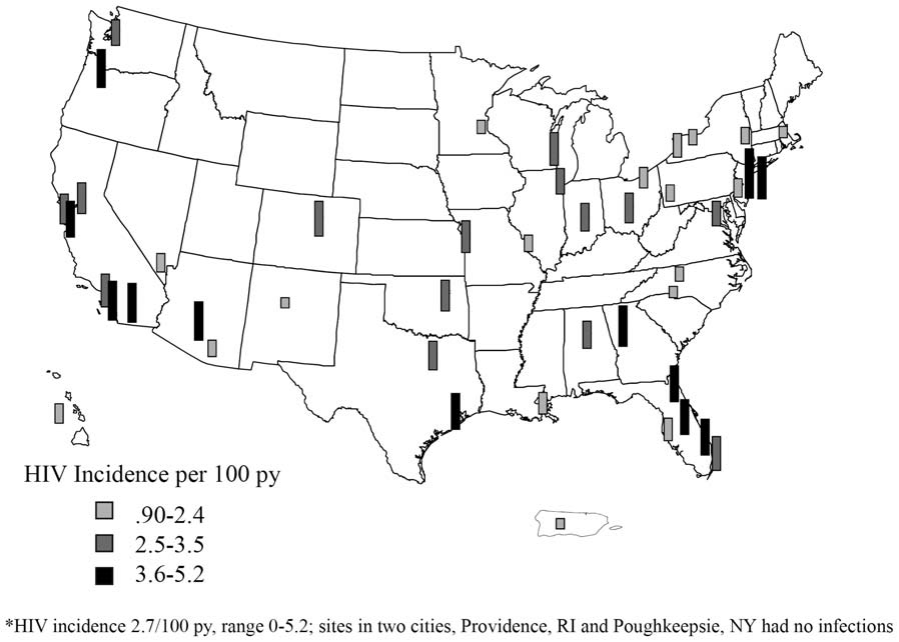
HIV incidence among MSM study participants in the United States, by city, 1998–2002*.

Overall, HIV seroincidence was 2.7/100 py (95% CI, 2.5, 3.0) and was relatively consistent across study year (1998–99, 78 cases, 2.5/100 py, 2000, 137 cases, 3.2/100 py, 2001, 101 cases, 2.7/100 py, and 2002, 22 cases, 1.8/100 py [p = .05]). Incidence did not vary by geographic region or race/ethnicity (Table 1), but was substantially higher among young MSM (3.7/100 py; 95% CI 3.0, 4.5). Twenty-three cities had HIV seroincidence rates above 2.7/100 py; twelve cities exceeded 3.5/100 py (Figure 1). Although, HIV incidence was expectedly high among MSM from New York City and Denver, it was also high among MSM from Atlanta, Houston, Jacksonville, and Phoenix. HIV seroincidence was highest among MSM who reported receptive UAI with HIV positive or unknown partners at baseline, followed closely by any receptive UAI, and then any UAI with HIV positive or unknown male partners. Elevated HIV seroincidence rates were noted among men who reported baseline recreational drug use, especially amphetamines (5.0/100 py; 95% CI 3.8, 6.4) and crack (5.2/100 py; 95% CI 2.7, 8.8).

### Factors predictive of HIV seroconversion

In univariate analysis, young men, men who reported a STI, or recreational drug use were more likely to seroconvert (Table 2). Variables, such as education level, geographic region, and race/ethnicity, and treatment arm assignment (HR = 1.01; 95% CI 0.78, 1.31) were not significant. Sexual risk behaviors predictive of HIV infection included: multiple male sex partners, reporting ≥1 HIV positive male sex partners and reporting UAI with HIV positive or unknown male partners. Perceived vaccine efficacy and perceived treatment assignment were not predictive of HIV seroconversion, p = .47, and p = .62, respectively.

**Table 2:** Risk factors for HIV infection among MSM in univariate and multivariate analyses, 1998–2002.

Risk factor	HR (95% CI)	P	Adjusted HR (95% CI)	P
**Age (years)**		0.008		0.003
18–30	2.1 (1.3–3.5)		2.4 (1.4–4.0)	
31–40	1.7 (1.1–2.8)		1.6 (1.0–2.6)	
41–50	1.3 (.81–2.2)		1.3 (0.8–2.2)	
51–64	1.0		1.0	
**History of STI**				
No	1.0			
Yes	1.6 (1.0–2.4)	0.03	-	
**Sexual behavior**				
No. of male sex partners in the last 6 months		<.0001		<.0001
≤4	1.0		1.0	
5–10	2.2 (1.6–3.0)		1.8 (1.3–2.6)	
>10	3.6 (2.7–4.9)		2.4 (1.7–3.3)	
No HIV positive male sex partners	1.0		1.0	
≥1 HIV positive partner	2.1 (1.6–2.7)	<.0001	1.6 (1.2–2.0)	.001
No UAI with an HIV positive/unknown partner	1.0		1.0	
UAI with an HIV positive/unknown partner	2.9 (2.3–3.7)	<.0001	1.7 (1.3–2.3)	<.0001
**Recreational drug use**				
No amphetamines	1.0		1.0	
Amphetamines	2.9 (2.2–3.9)	<.0001	1.6 (1.1–2.1)	.007
No tranquilizers	1.0			
Tranquilizers	1.6 (1.2–2.2)	0.006	-	
No hallucinogens	1.0			
Hallucinogens	3.0 (2.7–3.3)	<.0001	-	
No poppers	1.0		1.0	
Poppers	2.7 (2.1–3.4)	<.0001	1.7 (1.3–2.2)	<.0001
No sexual performance enhancing drugs	1.0			
Sexual performance enhancing drugs	2.0 (1.5–2.6)	<.0001	-	
No cocaine	1.0			
Cocaine	1.7 (1.2–2.3)	0.002	-	

NOTE. Multivariate model controlled for race, treatment arm assignment, date of study entry, education level, and geographic region. CI, confidence interval; HR, hazards ratio; UAI, unprotected anal intercourse.

In the multivariate time-varying model, significant predictors of HIV seroconversion included younger age, reporting greater numbers of male sex partners, having at least one HIV-infected male sex partner, UAI with HIV positive or unknown male partners during the preceding 6 months, and amphetamine and popper use (Table 2). The following variables were included in the model but were not significant: date of study entry, race, education level, geographic region, and treatment arm assignment (aHR = 1.04; 95% CI 0.80, 1.36).

## Discussion

This analysis of data from a large HIV vaccine efficacy trial in U.S. MSM provides important information about the national HIV epidemic. Although a largely white population, over 70% of participants were enrolled from cities lacking substantial epidemiologic information derived from MSM HIV seroincidence cohorts [19]–[23]. It is noteworthy how consistent MSM HIV seroincidence was across U.S. cities.

From 1998–2002, HIV seroincidence was 2.7/100 py among this U.S. MSM cohort. It remained relatively constant over each year of observation despite intensive ongoing HIV counseling and testing and reported decreases in HIV sexual risk behaviors [24]. HIV seroincidence was not associated with geographic region, race/ethnicity, education level, and calendar year, but substantially higher incidence was observed among younger men and men who reported risky sexual behaviors, recreational drug use, or a STI history at baseline. Many of these items were confirmed in the time varying multivariate model which indicated that younger age, having a positive male partner, UAI with an HIV-infected or unknown serostatus male partner, multiple male sex partners, and amphetamine and popper use were all independently predictive of HIV infection.

Since this trial excluded men who reported current IDU, few reported its use, and it was not incorporated into the multivariate model. However, non-injection recreational drug use was extremely high. Over 60% of MSM reported recreational drug use at enrollment, and high HIV seroincidence was seen among those MSM. Amphetamine and popper use were significantly linked to higher risks of seroconversion. Although, this study did not collect data on circumstances of drug use, other studies have demonstrated sexual situation-specific use of recreational drugs among MSM [25]–[29]. The popularity of these drugs is attributed to their reported abilities to facilitate access to certain types of sexual partners, specific types of sexual exchange, and to improve sexual performance [29], [30]. Amphetamines, methamphetamine in particular, have been associated with unprotected intercourse among both HIV negative and HIV positive MSM [26], [27], [31]–[36], condom failure [37] and HIV infection [23], [25], [28], [38]. Given these findings, it was concerning that amphetamine use increased over the course of the trial, suggesting concurrent and continued expansion in MSM populations outside the trial and subsequent increases in HIV incidence, especially among young users.

Seroincidence among young men was much higher compared to older MSM, and younger age was a significant predictor of HIV seroconversion. These data confirm results from other studies that young MSM are at greater risk for HIV seroconversion [19], [39]–[41] perhaps due to riskier sexual activities [11], [19], [42], partner choice [43], [44], internet-facilitated higher risk sexual encounters [45]–[49] and psychosocial attitudes [42], [50]. Without targeted prevention activities directed at this group, HIV seroincidence will continue to remain elevated among young MSM.

This analysis had several limitations. The study sample, although regionally diverse, was not representative of MSM at the state or national level and was not a population-based cohort. Generalizability is limited since minority men, younger men, and less educated men, made up smaller components of the study population, and were also less likely to be retained in follow-up. The study population also reflects only MSM interested in participating in an HIV vaccine trial. Study eligibility criteria were based on vaccine preparedness studies designed to enroll a high-risk HIV-negative MSM population sufficiently powered to detect a vaccine efficacy against sexual transmission and excluded men with a history of IDU or in monogamous relationships with HIV-negative men. Behavioral data were collected via an interviewer-administered questionnaire, thus socially undesirable drug using and sexual behaviors may have been underreported. Lastly, the lack of data regarding frequency, type, and circumstances of recreational drug use or the presence of primary or casual partners prevented in depth analysis of their contribution to seroconversion.

MSM have been a population long-affected by HIV and targeted for interventions by numerous prevention programs. However, despite awareness of their risk for HIV infection, HIV seroincidence continues to be elevated nationally among this group, especially among young men [1], [2], [4], [5], [14]. Although unsafe sexual risk behaviors were important predictors of HIV seroconversion and should be highlighted in any MSM prevention activity, our data and that of others reveal that drug use, in particular amphetamines and poppers, are also strongly associated with HIV infection. Most HIV risk reduction strategies concentrate largely on reducing sexual risk by encouraging condom use, reducing the number of sexual partners, promoting safer sexual practices, and stopping injection drug use [51]. Alcohol and other substance use which may be addressed in the overall safer sex guidelines may not be stressed as specific risk factors. The consistent drug use over the course of the study in the face of declining sexual risk behaviors [24] suggests that prevention messages delivered in the HIV counseling and testing of MSM may not adequately acknowledge or address all risk behaviors that place them at risk for HIV infection. Continuing development of promising new HIV prevention strategies [52], [53] and intensification of existing effective interventions (e.g. frequent repeat HIV testing and early linkage to care and treatment [54]–[56]) will be necessary to reduce the rate of HIV, especially among drug using young MSM.
